# Case report on a defective antibody response against pneumococcal serotype 9V in a patient with a single episode of pneumonia

**DOI:** 10.1186/s41479-017-0040-3

**Published:** 2017-11-05

**Authors:** Diana van Kessel, Thijs Hoffman, Heleen van Velzen-Blad, Bob Meek, Suzan van Mens, Jan Grutters, Ger Rijkers

**Affiliations:** 1 Department of Pulmonology and Respiratory Medicine, St. Antonius Hospital, Koekoekslaan 1, 3435 CM Nieuwegein, The Netherlands; 2 Laboratory of Medical Microbiology and Immunology, St. Antonius Hospital, Koekoekslaan 1, 3435 CM Nieuwegein, The Netherlands; 3 Division of Heart and Lungs, University Medical Center, Heidelberglaan 100, 3584 CX Utrecht, The Netherlands; 4 Department of Science, University College Roosevelt, Lange Noordstraat 1, 4330 AB Middelburg, The Netherlands; 5 Department of Pharmaceutical Sciences, Utrecht University, Utrecht, The Netherlands

**Keywords:** Community-acquired pneumonia, *Streptococcus Pneumoniae*, Antibody deficiency, Vaccination, Polysaccharide vaccine

## Abstract

**Background:**

Patients with recurrent respiratory tract infections and an impaired response to pneumococcal polysaccharide vaccination are diagnosed with a specific antibody deficiency. In adult patients with pneumococcal pneumonia an impaired antibody response to the infecting pneumococcal serotype can sometimes be found. It is unknown whether these patients are unable to produce an adequate anti-polysaccharide antibody response to pneumococcal vaccination after recovery.

**Case presentation:**

The authors describe a case of invasive pneumonia caused by *Streptococcus pneumoniae* serotype 9V in a previously healthy 35-year-old female. This patient did not produce serotype-specific antibodies against the infecting serotype during disease. After pneumococcal polysaccharide vaccination 3 months after recovery, she responded adequately to most other pneumococcal serotypes, but still had no response to the infecting serotype 9V. However, after 9 years (and prior to pneumococcal-conjugate vaccination) normal antibody levels against 9V were found. These antibody levels further increased after pneumococcal-conjugate vaccination.

**Conclusion:**

The authors believe that this case is the first description of a temporary deficient response to the infecting pneumococcal serotype in adults, while other reports with similar observations all involved children.

## Background

Community-acquired pneumonia (CAP) is a serious disease, most frequently caused by *Streptococcus pneumonia* [1]. Invasive and non-invasive pneumococcal disease has a high mortality risk, especially in the elderly patient with comorbidities [2]. Vaccination with a 23-valent pneumococcal polysaccharide vaccine (23vPPV) induces antibody production against the external polysaccharide capsule of the pneumococcus [3]. An impaired response to pneumococcal polysaccharide vaccination can be a risk factor for recurrent respiratory tract infections [4]. Vaccination with 23vPPV is part of the immunological screening in patients with recurrent respiratory tract infections [5]. Those patients with an impaired response to pneumococcal polysaccharides are diagnosed with a specific antibody deficiency [5].

Previously it was shown that a substantial proportion of patients with pneumococcal pneumonia did not show an antibody response to the infecting pneumococcal serotype, either during the clinical course of the disease or during convalescence [6, 7]. This raises the question whether these patients are able to mount a serotype-specific antibody response after vaccination with 23vPPV.

This case report describes a patient with pneumococcal pneumonia in whom the infecting serotype was identified. After recovery, an assessment of the humoral immune status was made, including analysis of the antibody response to pneumococcal polysaccharide vaccination.

## Case presentation

**Fig. 1:**
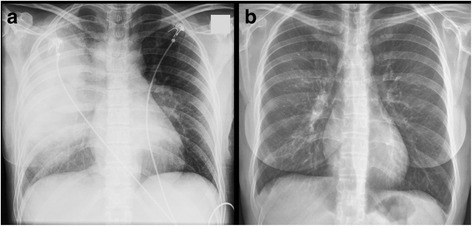
**a** Chest X-ray at time of presentation at the emergency department showing a large right-sided lobar infiltrate. **b** Chest X-ray at the outpatient department taken 42 days after initial presentation showing almost complete resolution of the infiltrate

A 35-year-old female was seen at the emergency department of St. Antonius Hospital, Koekoekslaan, The Netherlands. She presented with fever up to 40 degrees Celsius, shaking chills, dry cough, nausea, headache and right-sided chest pain. These symptoms were present for 1 week. The patient’s medical history was unremarkable; she didn’t use any medication and was a non-smoker. The diagnosis of pneumonia was made by physical and laboratory examination. The chest radiography showed a large right-sided lobar infiltrate (Fig. 1).

Because of impending respiratory insufficiency and hypotension, the patient was admitted to the intensive care unit and immediately intubated. Treatment with penicillin and erythromycin was started.

*Streptococcus pneumoniae* was detected as the causative microorganism by a blood and sputum culture and positive urine antigen testing, and identified as serotype 9V. A pharyngeal swab showed no signs of respiratory viruses on PCR testing. Antibiotic treatment was converted to penicillin only.

Following 9 days of mechanical ventilation, she was transferred to the ward where she made a good recovery and was discharged after 16 days.

Antibody titers against the capsular polysaccharides of 14 pneumococcal serotypes were measured on a multiplex immunoassay (Luminex 200, Luminex Corporation, Austin, Texas, United States [US]) as previously described (serotypes 1, 3, 4, 6B, 7F, 8, 9 N, 9V, 12F, 14, 18C, 19A, 19F, and, 23F; Danish nomenclature) [6]. Samples were taken at the day of admission and 42 days later. In the recovery sample the patient showed a low antibody titer and no titer rise against pneumococcal serotype 9V. She had not been vaccinated with a pneumococcal vaccine in the past.

Three months after admission to the hospital, the patient was seen at the outpatient department for a work-up according to the diagnostic protocol developed by the European Society for Immunodeficiency [5]. In that context, 23vPPV was administered. Pneumococcal polysaccharide antibodies against 8 pneumococcal serotypes were measured on a Luminex platform before vaccination and 4 weeks after vaccination (serotypes 3, 4, 6B, 9V, 14, 18C, 19F, and 23F; Danish nomenclature). The results showed that the patient had a sufficient antibody response to 5 of the 8 pneumococcal serotypes tested (Table 1), but no response whatsoever against her infecting serotype 9V. The patient therefore did not have an adequate antibody response specifically to pneumococcal serotype 9V either after natural exposition or after vaccination with 23vPPV. Immunological work up showed no other abnormalities; i.e. serum immunoglobulins, IgG-subclasses and complement were all normal (Table 2). The patient has had no severe or recurrent infections during the 9 years that have followed this first episode of pneumonia.

**Table 1:** Pneumococcal antibodies (μg IgG/ml) during pneumonia and after pneumococcal polysaccharide (conjugate) vaccination

	Community-acquired pneumonia		23 V Pneumococcal polysaccharide vaccination (+ 3 months)		13 V Pneumococcal conjugate vaccination (+10 years)	
Timeline	Jan–Feb 2005		May–June 2005		July–August 2014	
Pneumococcal serotype	Hospital admission (day 1)	Recovery (day 42)	Pre-vaccination	Post-vaccination	Pre-vaccination	Post-vaccination
3	0.12	0.14	0.05	2.43	6.27	13.09
4	0.06	0.03	0.15	0.17	0.02	0.42
6B	0.34	0.33	0.05	0.28	2.63	42.76
**9V**	**0.43**	**0.25**	**0.08**	**0.06**	**4.06**	**15.35**
14	2.25	2.67	3.21	7.28	6.91	9.55
18C	3.41	4.23	3.76	49.84	39.80	37.41
19F	1.03	1.08	0.60	6.85	31.96	77.10
23F	0.47	0.55	0.47	2.13	1.55	17.29

A sufficient serotype-specific response is defined as having titers higher than 1.3 μg/ml and at least a two-fold increase between pre- and post-vaccination titers [4]

Antibody levels to the infecting pneumococcal serotype (9V) are indicated in bold

**Table 2:** Laboratory results of immunological work-up, performed 3 months after recovery from the episode of pneumonia

Immunoglobulins	IgM (g/l)	1.01
	IgG (g/l)	11.6
	IgA (g/l)	2.19
	IgG1 (g/l)	6.9
	IgG2 (g/l)	3.4
	IgG3 (g/l)	0.4
	IgG4 (g/l)	0.5
Specific IgG antibodies	EBV VCA	pos
	CMV	neg
	Toxoplasma	neg
	Rubella	pos
Isohaemagglutinins	Anti-A	1/32
Complement	CH50 (%)	95
	AP50 (%)	75
	MP (%)	90

Recently the patient was included in the so-called CAPolista study (patient #6) [8]. In this study, patients who have experienced an episode of community-acquired pneumonia were vaccinated with 13-valent pneumococcal conjugate vaccine and antibodies were measured before and after vaccination. Antibodies were measured on a Luminex platform as previously described (serotypes 1, 3, 4, 5, 6A, 6B, 7F, 9V, 14, 18C, 19A, 19F, 23F; Danish nomenclature) [8]. Quite unexpectedly, a high anti-9V titer of 4 μg IgG/ml was found in the pre (conjugate) vaccination serum, which increased 3.8-fold after vaccination (Table 1). Also for serotype 6B the antibody titers had increased substantially in the 10 years after pneumococcal polysaccharide vaccination. For serotype 4 the antibody levels were and remained low (Table 1).

## Discussion and conclusions

Immunological investigation in this case of severe pneumonia in a previously healthy 35-year-old female showed an absent pneumococcal antibody response to the infecting serotype during the course of the disease. Upon vaccination with the 23vPPV 3 months after recovery, 9V antibodies remained low. Nine years later, a protective (i.e. >1.3 μg/ml) level of 9V antibodies was found prior to revaccination with the 13-valent conjugate vaccine, and this level increased further after conjugate vaccination.

According to the 2015 AAAAI/ACAAI criteria [4] this patient has an impaired response to pneumococcal capsular polysaccharides because she had an adequate response to less than 70% of the tested serotypes (5 out of 8). In the authors’ opinion, however, this patient does not meet the diagnostic criteria (inadequate response to pneumococcal polysaccharide vaccination, in combination with clinical characteristics suggestive of an immunodeficiency, such as recurrent infections [4]), because she had no recurrent respiratory tract infections before or after the episode of the pneumococcal pneumonia.

The high antibody titer against 9V (and 6B for that matter) 9 years after the invasive pneumonia caused by serotype 9V and 23vPPV vaccination indicates a temporary hyporesponsiveness to selective serotypes including the infecting serotype that recovered and high antibody levels were observed after 9 years that further increased upon revaccination with the 13-valent conjugate vaccine. All serotype specific antibody measurements were repeated with essentially the same results (not shown). There are no additional blood samples from the 9-year period; the patient was in good clinical condition and didn’t report back at the hospital. Therefore, it is unknown when the 9V antibodies increased or whether there was renewed colonization or a subclinical infection with 9V during that period. At any rate, there was a temporary defect in the ability to respond to serotype 9V pneumococci. The cellular and/or molecular causes of this temporary hyporesponsiveness are unknown.

Serotype-specific hyporesponsiveness towards the infecting serotype has been described in children [9]. In children who had been vaccinated with PCV prior to developing invasive pneumococcal disease, antibody levels directly after the infection were lower against the infecting serotype compared to other vaccine serotypes [9]. Furthermore, it has been observed that some children with invasive pneumococcal disease remained hyporesponsive to the infecting pneumococcal serotype, even after recovery [9, 10]. A possible explanation for this phenomenon is that hyporesponsiveness to PCV can be caused by pneumococcal carriership at time of vaccination. It has been shown that nasopharyngeal colonization with a specific pneumococcal serotype at the time of vaccination is associated with a lower response to that serotype, even after subsequent booster vaccinations [11, 12]. Another possible explanation is that during the pneumococcal infection, a high load of circulating polysaccharide antigens can cause a temporary immune paralysis [13]. The above considerations have been made based on observations in children, and data in adults are lacking.

In this case, immune investigations were performed because the patient participated in a study on pneumococcal pneumonia in the authors’ center. This specific immunological defect would normally not have been found, as the patient did not meet the criteria for immunological screening [5]. This case gives rise to the debate whether all hospitalized patients with a first episode of pneumococcal pneumonia should undergo an immune status assessment, or at least pneumococcal vaccination and measurement of antibodies to the most common pneumococcal serotypes. The CAPolista study has shown that the vast majority of CAP patients who were vaccinated with a conjugated polysaccharide vaccine do show an adequate antibody response [8].

In conclusion, these findings suggest that temporary hyporesponsiveness towards the causative pneumococcal serotype in CAP patients may occur, but does not necessarily indicate a selective immunodeficiency because recovery of the impairment occurred over time. It is unknown whether the temporary impairment was the cause of pneumonia or occurred due to a high load of capsular polysaccharides because of this pneumonia. However, this case report illustrates that pneumococcal infections may be associated with temporary hyporesponsiveness to infecting serotypes in adults as well as children.
